# Exposure of *Salmonella enterica* Serovar Typhimurium to High Level Biocide Challenge Can Select Multidrug Resistant Mutants in a Single Step

**DOI:** 10.1371/journal.pone.0022833

**Published:** 2011-07-29

**Authors:** Rebekah N. Whitehead, Tim W. Overton, Caroline L. Kemp, Mark A. Webber

**Affiliations:** 1 Antimicrobial Agents Research Group, School of Immunity and Infection, University of Birmingham, Birmingham, United Kingdom; 2 Biochemical Engineering, School of Chemical Engineering, University of Birmingham, Birmingham, United Kingdom; Charité-University Medicine Berlin, Germany

## Abstract

**Background:**

Biocides are crucial to the prevention of infection by bacteria, particularly with the global emergence of multiply antibiotic resistant strains of many species. Concern has been raised regarding the potential for biocide exposure to select for antibiotic resistance due to common mechanisms of resistance, notably efflux.

**Methodology/Principal Findings:**

*Salmonella enterica* serovar Typhimurium was challenged with 4 biocides of differing modes of action at both low and recommended-use concentration. Flow cytometry was used to investigate the physiological state of the cells after biocide challenge. After 5 hours exposure to biocide, live cells were sorted by FACS and recovered. Cells recovered after an exposure to low concentrations of biocide had antibiotic resistance profiles similar to wild-type cells. Live cells were recovered after exposure to two of the biocides at in-use concentration for 5 hours. These cells were multi-drug resistant and accumulation assays demonstrated an efflux phenotype of these mutants. Gene expression analysis showed that the AcrEF multidrug efflux pump was de-repressed in mutants isolated from high-levels of biocide.

**Conclusions/Significance:**

These data show that a single exposure to the working concentration of certain biocides can select for mutant *Salmonella* with efflux mediated multidrug resistance and that flow cytometry is a sensitive tool for identifying biocide tolerant mutants. The propensity for biocides to select for MDR mutants varies and this should be a consideration when designing new biocidal formulations.

## Introduction

*Salmonella enterica* serovar Typhimurium is a major cause of gastrointestinal disease and, as with many bacteria, *Salmonella* infections are becoming harder to treat because a high percentage of isolates are now resistant to commonly used antibiotics. Due to this increasing prevalence of resistance in pathogenic isolates there is a greater than ever requirement for effective cleaning and disinfection regimes to prevent infections and contain outbreaks. These regimes rely on the effective use of biocides, there is concern that the resulting increased use of biocides in farming, food production, hospital settings and the home is contributing to the selection of antibiotic resistant strains as some mechanisms of biocide resistance also confer antibiotic resistance [1]. Biocides incorporate disinfectants, antiseptics and preservatives, compounds that are often composed of a mixture of ingredients that act upon a wide range of cellular mechanisms and targets. This wide target base makes it difficult for bacteria to become resistant to biocides [1]. However, biocides are not always used at the correct concentration and can become compromised by coming into contact with organic material [1]. Bacteria that survive a low level dose of biocide are more likely to be resistant to antibiotics [2]. A single exposure to some biocides has previously been found to be insufficient to select for multidrug resistant (MDR) strains [3], however, repeated, sub-inhibitory exposure to biocides does result in selection of MDR bacteria. The increased accumulation of biocides in the environment at low levels has the potential to provide environments which will favour the selection of mutants with increased tolerance to biocides and antibiotics [4].

Low-level biocide-antibiotic cross resistance can result from the increased expression of efflux pumps, particularly in Enterobacteriaceae the tri-partite AcrAB-TolC system [5], [6]. AcrAB-TolC is the major multidrug efflux pump in many Gram-negative bacteria, including *Salmonella*, and has been shown to be over-expressed in *Salmonella* in response to exposure to low doses of biocide [3], [7]. Non-specific, low-level resistance to antibiotics and biocides can also be mediated by decreasing permeability of the membrane often due to repression of major porins [8]. As well as its role in antimicrobial resistance, the AcrAB-TolC efflux pump also has a role in *Salmonella* pathogenicity, all three of the components being required for virulence [9], [10], [11]. *Salmonella*, as with all bacteria, has a large complement of transporters with nine known multidrug efflux systems [12]. Whilst AcrAB is the major system, expression of other RND pumps (AcrEF and AcrD) is co-ordinately regulated with AcrAB in *Salmonella* and AcrEF can functionally complement AcrAB [13]. AcrAB-TolC production is tightly regulated, three homologous global activators, MarA, SoxS and Rob, and one local repressor, AcrR, have been shown to regulate *acrAB* in *Escherichia coli* [14], [15], [16], [17], [18]. An additional activator, RamA, also homologous to MarA, SoxS and Rob, is involved in *acrAB* regulation in *Salmonella* but is absent in *E. coli* [19]. Over-expression of RamA leads to an increase in expression of both *acrAB* and *acrEF* [20], [21].

Flow cytometry (FCM) and fluorescence-activated cell sorting (FACS) are techniques that can investigate and separate individual bacteria from large populations depending on their light scattering and fluorescent properties. Fluorescent dyes can be used to monitor membrane integrity and potential and thus monitor bacterial physiology; a major advantage of the technique is that it does not rely upon bacterial growth on agar plates or in broth, and so is suitable for studying bacteria having a viable but non-culturable (VNC) phenotype. In this study we have utilised FCM to analyse the physiological response of the model food borne pathogen *Salmonella* to both low-level and “in-use” concentrations of biocides. Using FACS we isolated mutants able to survive challenge with “in-use” concentrations of two biocides after one exposure. These mutants were multidrug resistant and over-expressed the AcrEF efflux pump and MarA, demonstrating that biocide exposure can select for mutants with a broad, low-level antibiotic resistance.

## Results

### Inhibitory concentrations of biocides

The recommended in-use concentration for all the biocides tested is 1%, Virkon (V), an oxidative compound, was found to inhibit growth of *Salmonella* at 0.25% (v/v), Superkill (SK), a mixture of aldehydes and quaternary ammonium compounds (QACs) at 0.002%, AQAS (AQ), a quaternary ammonium compound, at 0.002% and Trigene (TR), a halogenated tertiary amine compound, at 0.0005%.

### Isolation of viable cells from low and in-use concentrations of biocide

**Figure 1:**
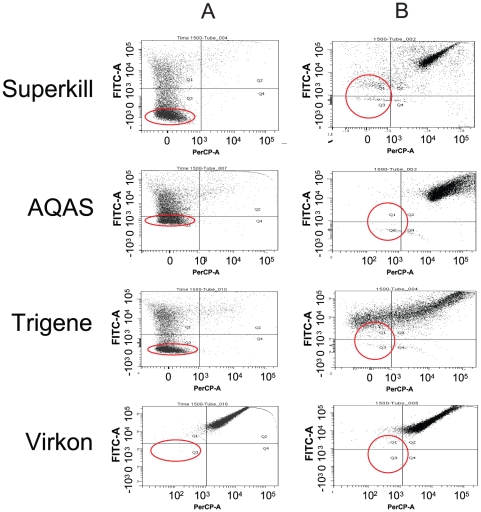
Analysis of the impact of biocide exposure on a population. Flow cytometry analysis of 10,000 *S.* Typhimurium cells after 5 hours challenge with a) low dose (SK 0.005%, AQ 0.005%, TR 0.002% and V 0.4%) and b) in-use concentration (1%) of 4 different categories of biocide. For each plot, the x-axis plots PI fluorescence and the y-axis BOX fluorescence. Quadrants represent: lower left, PI^−^ BOX^−^ therefore ‘healthy’; upper left, PI^−^ BOX^+^ therefore intact but depleted membrane potential (‘unhealthy’); upper right, PI^+^ BOX^+^ therefore disrupted membrane and membrane potential and likely to be dead. The circles indicate cells sorted for further investigation.

Exposure to biocide concentrations of 0.4% (V), 0.005% (SK), 0.005% (AQ) and 0.002% (TR) were used to provoke a “low” dose challenge of each biocide, where bacterial growth was inhibited but bacteria were not necessarily killed. Each biocide was also tested at the recommended working concentration of 1%, after 5 hours incubation in biocide each culture was analysed using FCM and PIX staining (Figure 1). After low dose challenge, the biocides generated different physiological effects as revealed by FCM. The majority of cells in Superkill or Trigene were in the lower left (“healthy”) quadrant, 74% and 60% respectively, the majority of the remaining cells being in the upper left quadrant (disrupted membrane potential but not ruptured membrane). The biocide AQAS had a greater antimicrobial effect and only 39% of cells remained in the “healthy” quadrant. Virkon had a much more pronounced effect with only 0.3% of cells in the “healthy” quadrant after a low dose exposure, the majority of cells in the upper right “dead” quadrant. The healthiest cells in each population were sorted by FACS (Figure 1a), each sorted population gave rise to viable colonies on LB agar which were retained for further investigation.

After an “in-use” dose challenge the majority of cells were in the upper right “dead” quadrant (V 98%, SK 88%, AQ 98%, TR 70%) (figure 1b). These cells were stained by both PI and BOX, therefore had ruptured membranes, no membrane potential and were presumed dead. Surprisingly there were cells in each biocide not stained by PI or BOX, maintaining an intact membrane/membrane potential, present at a low frequency. These populations (circled) were sorted, cells sorted from Virkon and AQAS gave no culturable colonies but cells sorted after high-level Superkill and Trigene treatment were recovered and representatives retained (referred to as SK 1% and TR 1%).

### Colonies isolated from in-use concentrations of biocide have reduced susceptibility to multiple antibiotics but not to biocides

The colonies isolated from the low dose of biocide had an antibiotic susceptibility profile identical to SL1344. The two mutants isolated from in-use concentrations of Superkill and Trigene had a broad, low-level reduced susceptibility to multiple antibiotics, nalidixic acid (4- fold), chloramphenicol (3-fold), tetracycline (2-fold), and ciprofloxacin (2-fold) (Table 1). They also had a 2-fold reduced susceptibility to the biocide triclosan.

**Table 1:** Susceptibility of biocide tolerant mutants to antibiotics.

	MIC (µg/ml)					
	Nal	Chl	Tet	Kan	Tric	Cip
SL1344	2	1	0.5	4	0.06	<0.015
SK 1%	32	8	2	4	0.25	0.06
TR 1%	32	8	2	8	0.12	0.06
SK 1% *acrEF::cat*	8	N/A	0.5	4	0.12	<0.015
TR 1% *acrEF::cat*	8	N/A	0.5	4	0.12	<0.015

Nal = nalidixic acid, Chl = chloramphenicol, Tet = tetracycline, Kan = kanamycin, Tric = triclosan, Cip = ciprofloxacin.

Populations of healthy cells recovered after exposure to a low dose of biocide were challenged again with the same level of biocide and re-analysed by FCM. The percentage of cells present in the lower-left “healthy” quadrant was not significantly different to that of SL1344 when first challenged. This suggests that the potential to survive exposure to a low dose of biocide is randomly distributed amongst a population, reflecting heterogeneity in terms of growth phase and/or gene expression amongst the population; cells able to survive a single exposure to a low biocide concentration were not pre-disposed to biocide survival upon repeat challenge. The mutants recovered from the in-use biocide challenge were however stably multidrug resistant although they showed no increase in MIC of the biocide from which they were recovered.

### Biocides have varying effects on cell growth rate

**Figure 2:**
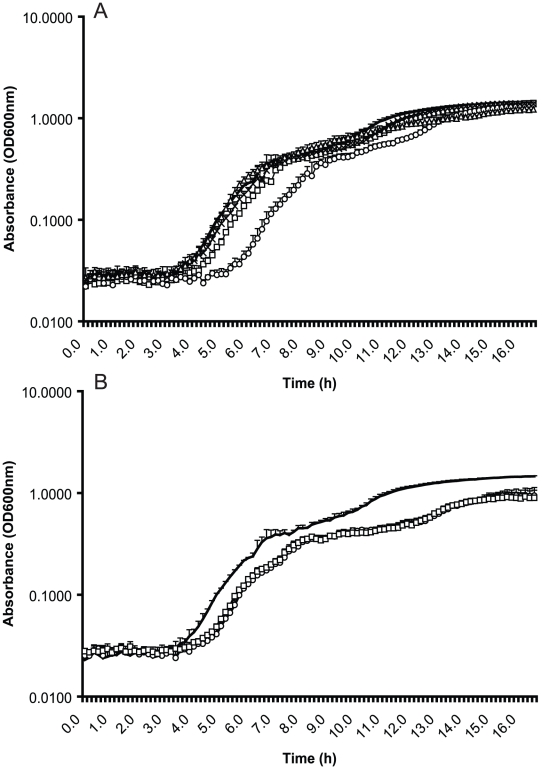
The impact of biocide exposure on growth. Panel A. Growth kinetics of SL1344 and cells sorted from exposure to a low dose of biocide concentration. The cells selected from Superkill show a slightly increased lag phase but grow to the same final OD_600_ as the WT. Lines show a mean of 3 technical replicates and are representative of the cells isolated from each biocide. Error bars are standard deviations of the technical replicates. SL1344 is shown by a continuous line, Superkill by a circle, AQAS by a triangle, Virkon by a square and Trigene by a cross. Panel B. Growth kinetics of SL1344 and mutants sorted from high-level biocide concentrations. The mutants have a much longer lag phase and fail to reach the same final OD_600_ as the WT. Lines show a mean of 3 biological replicates with the standard deviation. SL1344 is shown by a continuous line, SK 1% by a circle and TR 1% by a square.

Cells sorted from low dose challenge with Virkon and Trigene grew at a similar rate to wild-type cells in broth when no biocide was added (Figure 2a). Cells sorted from low dose challenge with Superkill had a slightly longer lag phase than wild-type cells and cells sorted from AQAS did not achieve as high an optical density after the same length of time. The mutants sorted from in-use concentrations of Superkill and Trigene had a longer lag phase than SL1344 and only achieved a final OD_600_ of 0.9, significantly lower than the OD_600_ of 1.5 achieved by SL1344 after 16 hours growth (Figure 2b). This shows a fitness cost has been incurred by both mutants.

### Cells isolated from in-use concentrations of biocide have increased efflux activity

**Figure 3:**
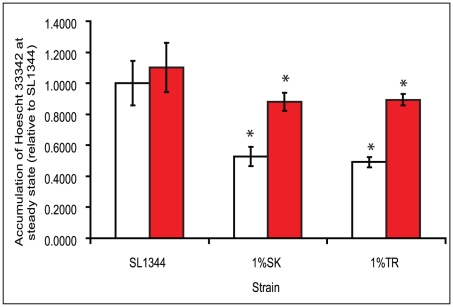
Efflux activity of biocide selected mutants. Relative levels of Hoescht accumulation by SL1344 and mutants recovered from high-level biocide exposure in the absence (white) and presence (black) of PAβN. Experiments were performed in triplicate, and the error bars indicate standard deviations. An asterisk indicates a statistically significant difference compared to the wild-type (*P*<0.05).

The efflux activity of the sorted cells was assessed by measuring intracellular accumulation of Hoescht 33342, a known substrate of MDR efflux pumps. The cells exposed to a low-dose of biocide accumulated levels of Hoescht dye similar to SL1344 (data not shown). The two mutants recovered from the in-use biocide challenge both accumulated significantly less dye than SL1344 (Figure 3) and in the presence of an efflux pump inhibitor PAßN, accumulation of Hoescht was elevated, confirming that these mutants have increased efflux activity compared with SL1344 (Figure 3).

### AcrEF and MarA are up-regulated in high level biocide mutants

**Figure 4:**
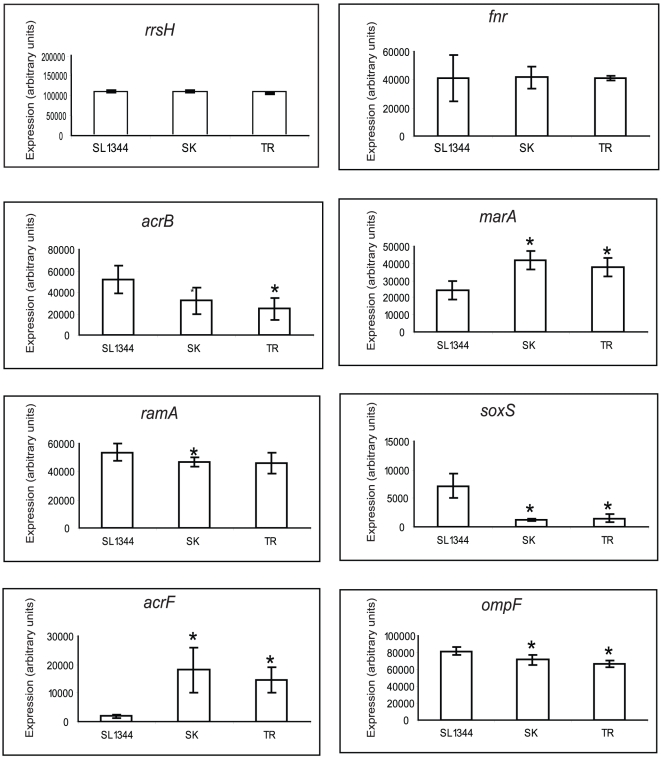
Expression of genes associated with multidrug resistance. Relative levels of gene expression by SL1344 and mutants recovered from high-level biocide exposure after growth to mid-logarithmic phase in LB broth. The data show the mean of 4 biological replicates and 2 technical replicates for each gene. Standard deviation and students ‘t’-test were used to validate the data and any significant difference in the mutants compared to SL1344 (*P*<0.05) is indicated by an asterisk.

Comparative RT-PCR was used to determine the level of expression of genes relevant to multidrug resistance in the high-level biocide mutants. In both mutants the expression of *ompF*, *soxS* and *acrB* was statistically significantly down-regulated in comparison with SL1344 (Figure 4). The *ramA* gene was significantly down-regulated in SK 1% but not TR 1%. The down-regulation of *acrB* in both mutants was a surprise as this is usually considered the major efflux system in *Salmonella*. In both SK 1% and TR 1%, *marA* (2 fold) and *acrF* (10 fold and 7 fold respectively) expression was however significantly increased compared to SL1344 (Figure 4) indicating that the efflux pump AcrEF-TolC is mediating the MDR phenotype in these mutants. Sequencing showed up-regulation of MarA was not due to a mutation in the *marA* promoter or within *marR*.

To confirm that AcrEF over-expression was responsible for the MDR phenotype seen in these mutants, we inactivated *acrEF* in these strains, this resulted in a broad loss of MDR (Table 1) thus confirming that the AcrEF pump is integral to the MDR phenotype seen. As *acrEF* has been reported to be regulated by FNR in *E. coli* [26] we investigated the level of *fnr* expression in our mutants but the level of expression was not significantly different between the mutants and wild-type.

### Effect of acrB, tolC and ramR mutations on SL1344 survival in low dose biocide

**Figure 5:**
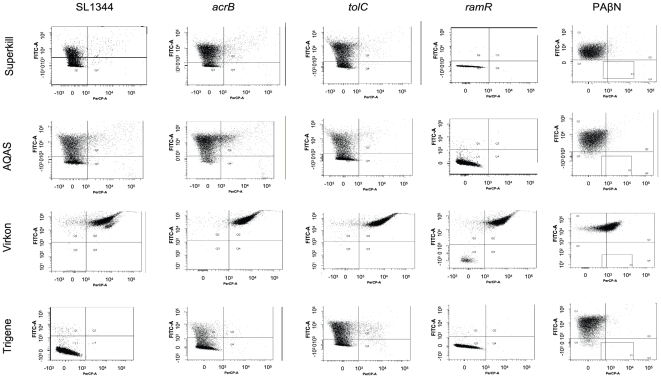
Impact of efflux on biocide survival. Flow cytometry analysis of the physiological state of 10,000 cells of SL1344, *acrB*, *tolC* and *ramR* isogenic mutants after a 5 hour exposure to a low dose of four different biocides. The final column shows the effect of addition of PAβN (100 µg/ml) on SL1344. The x-axis plots PI fluorescence and the y-axis BOX fluorescence.

To further explore the role of efflux in biocide tolerance a series of isogenic mutants (table 2) lacking components of AcrAB-TolC or over-expressing this system were examined for their ability to survive biocide exposure. The AcrAB-TolC system was studied as this is constitutively expressed whereas AcrEF is cryptic in wild-type cells. After 5 hours incubation in low dose biocide, the physiology of the wild-type strain SL1344 and isogenic mutants lacking a functional *acrB*, *tolC* and *ramR* were assessed by FCM (Figure 5). The striking result was that the *ramR* mutant (which over-expresses AcrAB-TolC) had a significantly greater proportion of cells in the lower-left “healthy” quadrant than SL1344 for all 4 of the biocides tested. The biggest effects were seen with Superkill and AQAS where percentages in the lower-left “healthy” quadrant for SL1344 were 5% and 36% respectively whereas for the *ramR* mutant 98% of cells in each biocide were in this quadrant. In each case, the wild-type had a larger proportion of BOX+ bacteria, lacking membrane potential, than the *ramR* mutant. A smaller but still consistent effect was seen for the Trigene and Virkon biocides where the *ramR* mutant had higher “healthy” percentages, 99% compared to 95% in Trigene and 1.8% to 0.2% in Virkon, where the majority of the remaining bacteria were in the upper-right “dead” quadrant. Virkon in particular seemed not to be affected by efflux pump loss or over-expression; this was also suggested by the addition of the efflux pump inhibitor (EPI) PAβN, which decreased the percentage of cells of SL1344 in the lower-left quadrant for all biocides bar Virkon (Figure 5).

**Table 2:** Strains used in this study.

Strain	Description	Origin/reference
SL1344	Wild-type	[24]
L109	SL1344, *tolC::aph*	[9]
L110	SL1344, *acrB::aph*	[9]
L1007	SL1344, *ramR::aph*	[27]
SK1	Mutant of SL1344 derived after superkill treatment	This study
TR1	Mutant of SL1344 derived after trigene treatment	This study
EG16568	SL1344, *acrEF::cat*	[28]
SK1-*acrEF*	SK1, *acrEF::cat*	This study
TR1-*acrEF*	TR1, *acrEF::cat*	This study

## Discussion

Successful prevention and eradication of outbreaks of bacterial infection relies on biocide use, as a result biocides must be able to rapidly and completely kill large populations of bacteria. Previously we have shown that repeated exposure of *Salmonella* to low levels of biocide can select for multidrug resistant mutants, in our previous work we were unable using conventional culture based techniques to isolate mutants with increased biocide tolerance. In this study we show that FCM is a very useful technique for identifying populations of bacteria that can withstand biocide challenge; FACS has the added advantage of allowing the physical isolation of those biocide-resistant bacteria for further investigation. The fact that FACS succeeded in isolating resistant mutants where plate-based screening failed suggests that the FACS method aids bacterial survival in this context; possibly as a result of the decoupling of the selection and growth processes.

Using this approach we were able to show that a single, low-level exposure to 2–4X the MIC of biocide, whilst preventing growth in broth did not rupture the membranes of a large proportion of a population (with the exception of Virkon) and that sorted ‘healthy’ cells were not MDR. This supports the previous findings that more than one exposure to a low level of biocide is required to select antibiotic resistant mutants. This might reflect the fact that most cells can survive this low-level stress (even if not able to grow) and that the natural variation within a population includes cells relatively unaffected by low-level biocide exposure. Therefore there is little pressure for selection of mutants with increased resistance. The flow cytometer was however also able to identify and recover mutants from populations that had been exposed to an extreme 1% biocide challenge. Subsequent, replicate experiments with traditional culture methods did not recover live mutants after incubation with 1% biocide. This indicates the potential for FACS to identify and select mutants that occur at a very low frequency.

Typically biocides are expected to provoke a 5-log reduction in viable bacterial numbers; in our experiments the proportion of cells in a population that were able to maintain a membrane potential after 5 hours exposure to the working concentration of biocide was 2% in Virkon and AQAS, 12% in Superkill and 30% in Trigene. After sorting, the vast majority of these populations did not grow and viable colonies were only recovered from Superkill and Trigene. However, we made no efforts to improve this recovery rate with the addition of a richer recovery medium than LB. The isolation of some immediately viable cells from a larger population of cells that maintain a membrane potential might suggest this population is highly heterogeneous and comprises efflux mutants as characterised here and possibly a larger viable but non-culturable population. Further work to investigate this is warranted.

The two resistant mutants characterised had a classic, low-level MDR phenotype with a slightly increased resistance to a broad range of drugs from different classes of antibiotics. The AcrAB-TolC efflux pump has been shown to be the most important efflux pump in expelling antibiotics from *Salmonella*. It was therefore expected that expression of this pump would be up-regulated in our recovered cells. However, the down regulation of *acrB* and the up-regulation of *acrF* indicate that the AcrEF-TolC efflux pump is the main driver in the MDR phenotype of these two mutants. This was confirmed by inactivation of AcrEF-TolC in these strains. Previous work has indicated a relationship between AcrAB-TolC and AcrEF-TolC; over-expression of AcrEF-TolC can complement AcrB deletion [13]. The fact that both the mutants recovered from two different biocides had this phenotype suggests a common mechanism of cell survival to biocides with de-repression of AcrEF (mediated by up-regulation of MarA) favoured over de-repression of AcrAB. The MarA regulon has not been shown to include *acrEF* in *Salmonella;* however in *E. coli* a putative MarA binding site in the *acrEF* promoter has been described [22]. Study of the *S*. Typhimurium *acrEF* promoter region confirms that this putative MarA binding site is also present and is a better match, by 1 base, to the putative binding site consensus sequence. This suggests that AcrEF de-repression in the mutants is a result of MarA over-expression. Sequencing of the mutant's *marA* promoter region showed no mutations. The genetic basis for *marA* de-repression in the mutants studied here is therefore currently unknown.

Of the isogenic mutants tested for ability to survive a low dose of biocide, only a *ramR* mutant had a discernable effect on the survival of the cells. This mutant is MDR and over-expresses AcrAB-TolC [23]. This suggests therefore that AcrAB-TolC can recognise some biocides as substrates (excluding Virkon) and that de-repression of AcrAB-TolC will be protective against biocide stress. Surprisingly, loss of *acrB* or *tolC* did not have a very detrimental effect on the cells. This might indicate that other efflux systems can complement the loss of the AcrAB-TolC pump. Chemical inactivation of efflux with PAβN also decreased the survival of the cells in Superkill, AQAS and Trigene. This again suggests that efflux is an intrinsic mechanism by which biocides are expelled from the cell; once again cells grown in Virkon were less affected by the addition of PAβN; more evidence efflux is of little importance in the elimination of this biocide.

This study has shown that *Salmonella* can survive challenge with in-use concentrations of some biocides, that this is due to de-repression of the AcrEF MDR efflux system and that these mutants are MDR. Flow cytometry is a sensitive tool for evaluating the cidal potential of biocides which offers significant advantages over culture based methods. Our work suggests the potential for different biocides to select for MDR varies; the development of novel biocide formulations should bear this in mind.

## Materials and Methods

### Isolation of biocide tolerant mutants

*Salmonella enterica* serovar Typhimurium strain SL1344 [24] was the parent strain used in all experiments, for biocide exposure overnight cultures (5 ml) of SL1344 were grown in LB broth at 37°C and used to inoculate fresh 5 ml LB broths containing the desired concentration of different biocides. After 5 hours samples were removed and analysed by FCM. An ethanol killed sample of bacteria was used as a control. For each experiment 10,000 cells were analysed and fluorescence from two dyes, PI (stains the DNA of bacteria that have lost membrane integrity) and BOX (stains bacteria with a collapsed membrane potential) used to identify cells with maintaining a membrane potential which were sorted by FACS into FACSFlow buffer and aliquoted onto LB agar plates. Colonies that grew were stored on Protect Beads (Technical Service Consultants Ltd). Full details of the methodology are given in Material S1.

### Phenotypic characterisation of biocide tolerant mutants

Cells recovered after biocide exposure were characterised in terms of their antimicrobial susceptibility using broth micro-dilution and agar dilution methods following the recommendations of the British Society for Antimicrobial Chemotherapy [25]. The growth of sorted biocide tolerant mutants compared to the parental strain SL1344 was also analysed measuring the optical density at 600 nm of replicate cultures. Full details of the methodology are given in Material S1.

### The role of efflux

Efflux activity was determined phenotypically by measuring the accumulation of Hoescht 33342, a substrate of MDR efflux systems according to the method of Webber & Coldham [26]. Accumulation of Hoescht 33342 was measured in the presence and absence of PAβN, an efflux pump inhibitor, added to a final concentration of 100 µg.ml-1. Expression of efflux pump genes *acrB*, *acrEF*, known regulators *marA*. *soxS*, *ramA* and *fnr*, porin *ompF* and 16S rRNA (*rrsH*) was also determined using *c*omparative RT-PCR as previously described [13]. Primers and full details are listed in Table S1. To confirm its role in multidrug resistance, the *acrEF* locus was disrupted in the SK 1% and TR 1% mutants using bacteriophage P22. Full details of the methodology are given in Material S1.

## Supporting Information

Material S1 **Full description of methodology.**
(DOC)

Table S1 **Primers used in this study.**
(DOC)
