# Supplementary material for: Exposure of Salmonella enterica Serovar Typhimurium to High Level Biocide Challenge Can Select Multidrug Resistant Mutants in a Single Step
Source: PLoS One. 2011 Jul 29;6(7):e22833. doi: 10.1371/journal.pone.0022833 (PMC3146503; doi:10.1371/journal.pone.0022833)
Supplement: Material S1 — Full description of methodology. (DOC) [file pone.0022833.s001.doc]

**Material S1**

*Antibiotics, Biocides and Dyes.* Antibiotics were all purchased from Sigma-Aldrich (Poole, UK) except ciprofloxacin from Fluka (Buchs, Switzerland), triclosan was a kind gift from Ciba Geigy (UK). Virkon and AQAS were from DuPont animal health, UK, Superkill from AFS Animal Care, UK and Trigene from Medichem International, UK. Propidium iodide (PI) and Bis (1,3-dibarbituric acid) trimethine oxanol (BOX) were both purchased from Sigma-Aldrich (Poole, UK). PI was made up in water to a stock of 200 µg.ml^-1^ and stored at 4 ^o^C. This was diluted to a working concentration of 5 µg.ml^-1^ in Dulbecco’s PBS (Gibco, UK). BOX was made up in DMSO (Sigma) to a stock of 10 mg.ml^-1^ and stored at -20 ^o^C. This was diluted to a working concentration of 10 µg.ml^-1^ in Dulbecco’s PBS. 100 µl of 4 mM EDTA (BDH, UK) was added to 9.9 ml of working concentration BOX to aid staining. Both PI and BOX working stocks were made on the day of use and sterilised by passing through a 0.22 µM filter (Millipore, UK). Bis-benzimide (Hoescht-33342) was purchased from Sigma and used at a working concentration of 25 µM.

*Flow cytometry and cell sorting.* *Salmonella enterica* serovar Typhimurium strain SL1344 [24] was the parent strain used in all experiments. Overnight cultures (5 ml) of SL1344 were grown in LB broth at 37 ^o^C and used to inoculate fresh 5 ml LB broths containing the desired concentration of different biocides with approximately 10^7^ cfu/ml. After 5 hours, 1 ml of culture was removed, pelleted by centrifugation and re-suspended in 500 µl of Dulbecco’s PBS. An ethanol killed sample of bacteria was used as a control (made by pelleting 1 ml of cells then re-suspending in 500 µl of 100 % ethanol for 10 minutes before pelleting again and resuspending in 500 µl PBS). To prepare the cells for the flow cytometer, 50 µl of the PBS-suspended cells were added to 1 ml of FACSFlow buffer (BD, UK) in a tube. PI and BOX were added and samples analysed with a FACS ARIA II (BD, UK). Cells were illuminated with a 488 nm laser and data from 10,000 particles were collected. Forward- and side-scatter data were collected along with PI fluorescence (red, collected through an LP 565 mirror and BP 610/20 filter) and BOX fluorescence (green, collected through an LP 502 mirror and BP 530/30 filter). PI stains the DNA of bacteria that have lost membrane integrity whilst BOX stains bacteria with a collapsed membrane potential. Data were analysed with FACSDiva (BD); post-collection compensation was used to eliminate spectral overlap and was calibrated using singly-stained ethanol-killed cells. Data are plotted using biexponential scaling. Particles smaller than bacteria were eliminated by adjusting the threshold values on FSC and SSC channels. Cells for further analysis were sorted into FACSFlow buffer and aliquoted onto LB agar plates. Colonies that grew were stored on Protect Beads (Technical Service Consultants Ltd).

*Susceptibility testing.* Both broth micro-dilution (to identify inhibitory concentrations of the different biocides) and the agar dilution (for antibiotics) methods were used following the recommendations of the British Society for Antimicrobial Chemotherapy [25].

*Analysis of growth kinetics.* The growth of sorted biocide tolerant mutants compared to the parental strain SL1344 was measured by inoculating approximately 10^4^ CFU/ml of an overnight culture into 200 µl of fresh LB broth. Samples were placed in triplicate in a microtitre plate and the optical density at 600 nm was measured every 10 min for 16 h in a FLUOstar OPTIMA at 37°C (BMG LABTECH, UK). Data was analysed using Excel (Microsoft, USA) to calculate means and standard deviation for each strain.

*Efflux accumulation assay.* Efflux activity was determined by measuring the accumulation of Hoescht 33342, a substrate of MDR efflux systems. The assay was according to the method of Webber & Coldham [26]. Accumulation of Hoescht 33342 was measured in the presence and absence of PAβN, an efflux pump inhibitor, added to a final concentration of 100 µg.ml^-1^.

*Analysis of gene expression.* RNA was extracted from 4 independent cultures of SL1344, SK 1% and TR 1% grown in LB broth to an OD_600_ of between 0.5 and 0.7. RNA was extracted using an initial phenol/ethanol stabilisation and the Promega RNA SV40 Kit. RNA was reverse transcribed to cDNA using Superscript III (Invitrogen). Comparative RT-PCR (cRT-PCR using dHPLC) was then used to measure expression of 16S rRNA (*rrsH*), *acrB*, *marA*, *ramA*, *fnr*, *acrEF*, *soxS* and *ompF* as previously described [13]. Primers are listed in supplementary material.

*Sequencing of the marA promoter.* The SK 1% and TR 1% *marA* promoter regions were sequenced to check for mutations. DNA was amplified from a single microlitre of a cell lysate using primers 423 and 424 (supplementary material), an annealing temperature of 60 ^o^C and an extension time of 2 minutes. The resulting products were sequenced in the Genomics Laboratory, University of Birmingham and the sequence compared to that of SL1344 (<http://www.genome.jp/tools/clustalw/>).

*Mutation of acrEF*. The *acrEF* genes were disrupted in the SK 1% and TR 1% mutants using bacteriophage P22. Briefly, phage was grown on donor *S*. Typhimurium EG16568 (*acrEF::cat)* [27] and then used to transduce the chloramphenicol-marked mutant allele into SK 1% and TR 1%. Transductants were selected on chloramphenicol-containing agar plates (25 µg/ml) and transfer of the mutant allele confirmed by PCR.
