# Supplementary material for: Exposure of Salmonella enterica Serovar Typhimurium to High Level Biocide Challenge Can Select Multidrug Resistant Mutants in a Single Step
Source: PLoS One. 2011 Jul 29;6(7):e22833. doi: 10.1371/journal.pone.0022833 (PMC3146503; doi:10.1371/journal.pone.0022833)
Supplement: Table S1 — Primers used in this study. (DOC) [file pone.0022833.s002.doc]

**Table S1. Primers used in all experiments.**

| **Gene**  **amplified** | **Experiment** | **ID Number (and orientation)** | **Sequence (5’ to 3’)** |
| --- | --- | --- | --- |
| *rrsH*  (16S) | Gene expression | 324 (forward)  325 (reverse) | CCT CAGCACATTGACGTTAC  TTCCTCCAGATCTCTACGCA |
| *acrB* | Gene expression | 334 (f)  335 (r) | CGTGTTATGACGGAAGAAGG  GCCATACCGACGACGATAAT |
| *ramA* | Gene expression | 542 (f)  543 (r) | TCCGCTCAGGTTATCGACAC  AGCTTCCGTTCACGCACGTA |
| *acrF* | Gene expression | 322 (f)  206 (r) | GACGTCCTATTTTCG  GATTATCTGAGACGAACC |
| *marA* | Gene expression | 326 (f)  327 (r) | CGCAACACTGACGCTATTAC  TTCAGCGGCAGCATATAC |
| *soxS* | Gene expression | 328 (f)  329 (r) | CATATCGACCAACCGCTA  CGGAATACACGCGAGAAG |
| *ompF* | Gene expression | 654 (f)  655 (r) | TGAAGGCGAACAGCAGAACT  ATATACGGCAGCCAGGTAGA |
| *fnr* | Gene expression | 1110 (f)  1111 (r) | GGTTGTGCTATCCATTGC  GGATCATATCCTGATCGC |
| *marA* | Promoter sequencing | 423 (f)  424 (r) | GCTGGATATCACCGCAACAC  GCGGACTTGTCATAGCCAGA |
| *acrF* | Verification of mutant | 205 (f)  206 (r) | CGGTTACTCAGGTTATCG  GATTATCTGAGACGAACC |
